# Supplementary material for: A Novel Deep Blue LE-Dominated HLCT Excited State Design Strategy and Material for OLED
Source: Molecules. 2021 Jul 28;26(15):4560. doi: 10.3390/molecules26154560 (PMC8348658; doi:10.3390/molecules26154560)
Supplement: Supplementary file 1 [file molecules-26-04560-s001.zip › molecules-1294290-supplementary.pdf]

molecules

Supporting Information

Article

A Novel Deep Blue LE-dominated HLCT Excited State Design Strategy and Material for OLED

Xuzhou Tian [1], Jiyao Sheng [2], Shi-Tong Zhang [1,*], Shengbing Xiao [1], Ying Gao [1], Haichao Liu [1] and Bing Yang [1,*]

[1] State Key Laboratory of Supramolecular Structure and Materials, College of Chemistry, Jilin University, 2699 Qianjin Street, Changchun, 130012, China; tianxz19@mails.jlu.edu.cn (X. Tian); xiaosb20@jlu.edu.cn (S. Xiao); yinggao19@mails.jlu.edu.cn (Y. Gao); hcliu@jlu.edu.cn (H. Liu)

[2] State Key Laboratory of Inorganic Synthesis and Preparative Chemistry, College of Chemistry, Jilin University, 2699 Qianjin Street, Changchun, 130012, China; shengjiyao@jlu.edu.cn (J. Sheng)

* Correspondence: stzhang@jlu.edu.cn (S. Zhang); yangbing@jlu.edu.cn (B. Yang)

Contents

S-I Figures

S-II Tables

S-III Illustrations

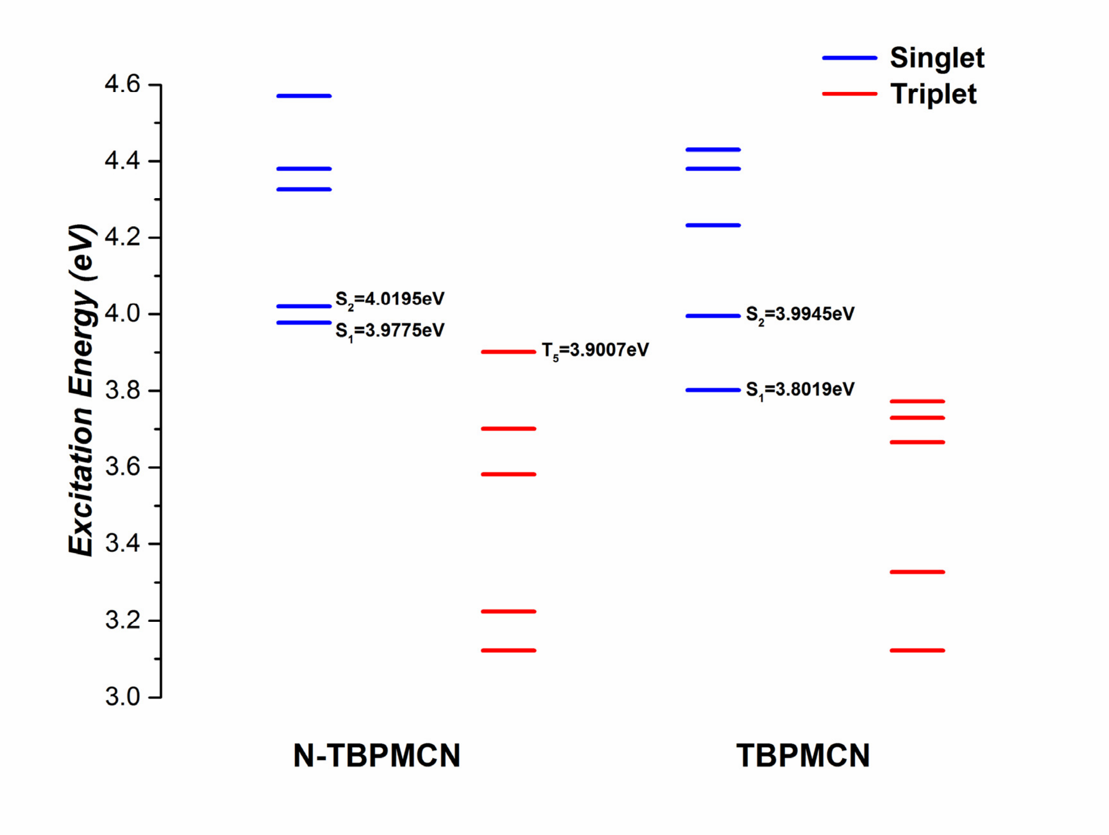

Figure S1. The energy landscape for excited states of **N-TBPMCN** and **TBPMCN**.

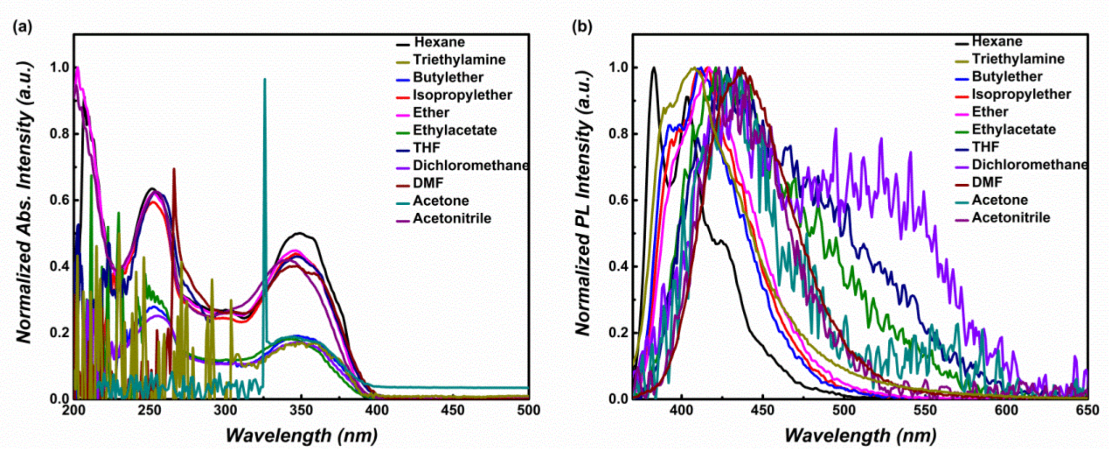

Figure S2. (a) The ultraviolet-visible (UV-Visible) absorption spectra of **N-TBPMCN**; **(b)** PL spectra of **N-TBPMCN** in diluted solutions (concentration = 1×10^{-5} mol·L^{-1}).

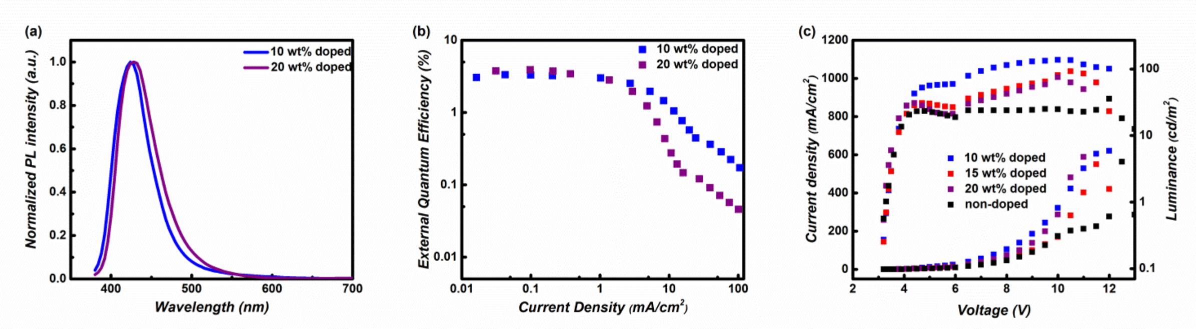

Figure S3. (a) The OLED EL spectra of **N-TBPMCN** device (10 wt% doped and 20 wt% doped); **(b)** the figure of EQE in different current densities (10 wt% doped and 20 wt% doped); **(c)** the current density-voltage-luminance (J-V-L) characteristic for 10 wt% doped and 20 wt% doped devices.

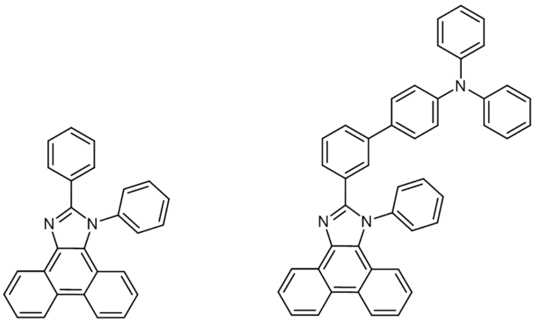

PPI **mTPA-PPI**

Figure S4. The structure of PPI and mTPA-PPI.

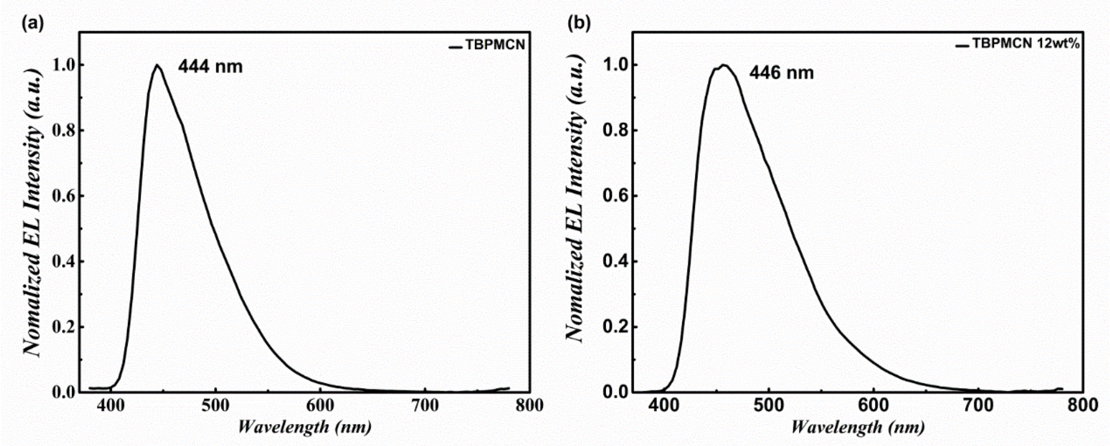

Figure S5. (a) The **TBPMCN** EL spectra of non-doped device; **(b)** the **TBPMCN** EL spectra of 12 wt% doped device.

(a)

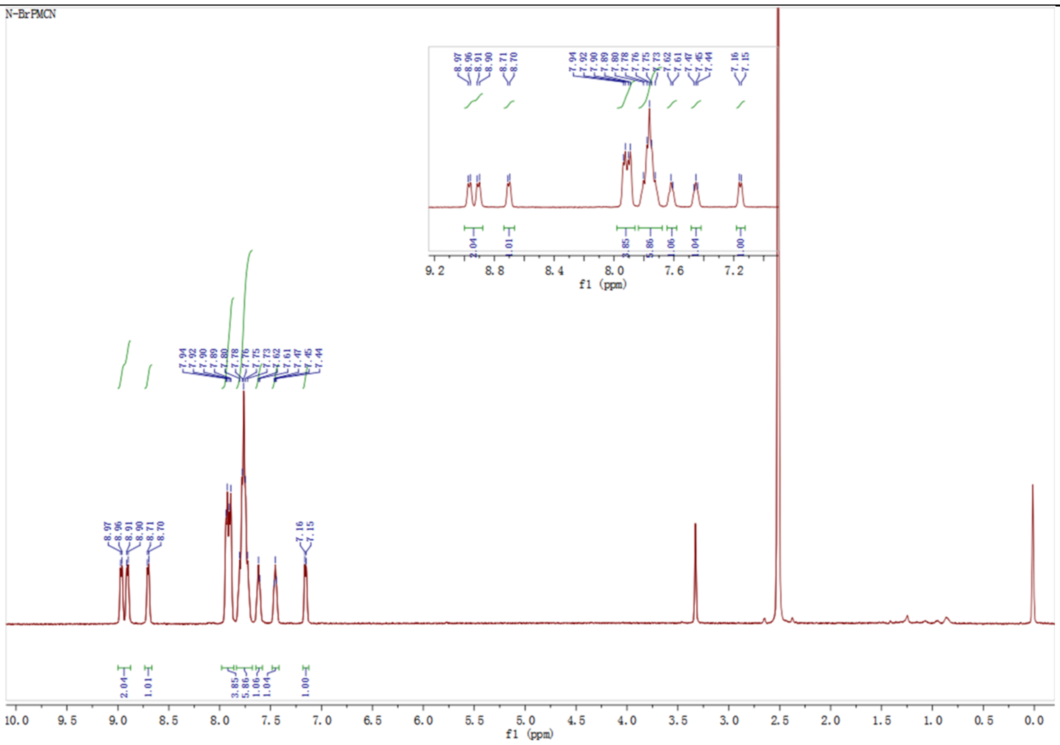

(b)

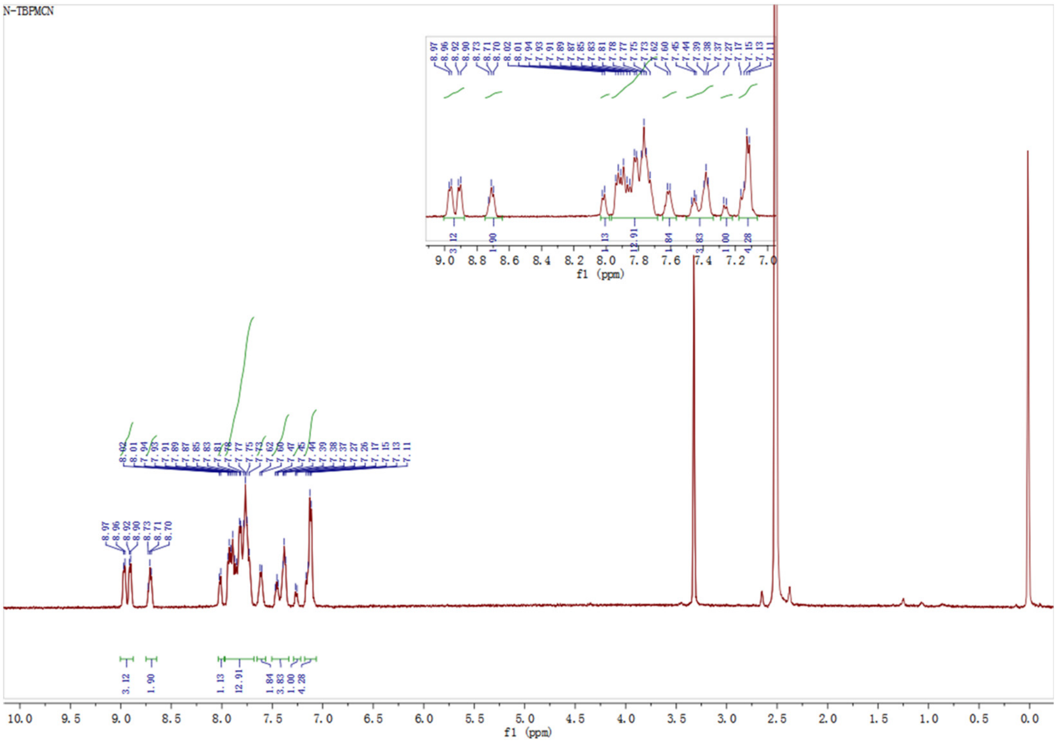

(c)

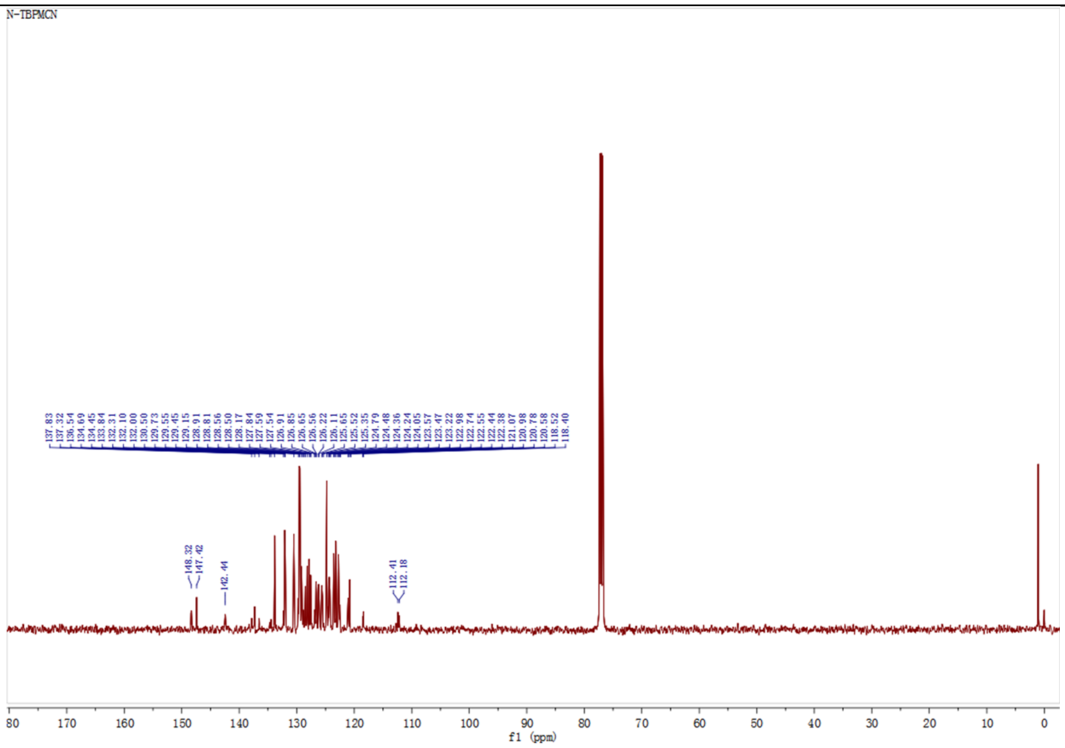

Figure S6. The ¹H NMR spectra of the **N-BrPMCN (a)** and **N-TBPMCN (b)**; the ¹³C NMR spectra of the **N-TBPMCN (c).**

S-II Tables

Table S1. The specific energy value of singlet and triplet for **N-TBPMCN** and **TBPMCN** calculated by density functional theory (DFT) method at a M062X/6-31g (d, p).

N-TBPMCN (eV)				N-TBPMCN (eV)			
**S_1**	**3.9775**	**T_1**	**3.1218**	**S_1**	**3.8019**	T_1	3.1218
S_2	4.0195	T_2	3.2242	S_2	3.9945	T_2	3.3271
S_3	4.3269	T_3	3.5819	S_3	4.2326	T_3	3.6657
S_4	4.3803	T_4	3.7009	S_4	4.3805	T_4	3.7283
S_5	4.569	T_5	3.9007	S_5	4.4296	T_5	3.7723
S_6	4.6401	T_6	3.9313	S_6	4.5396	T_6	3.8656
S_7	4.669	T_7	3.9666	S_7	4.558	T_7	3.9284
S_8	4.7193	T_8	4.0494	S_8	4.6657	T_8	3.9825
S_9	4.8828	T_9	4.221	S_9	4.801	T_9	4.1304
S_{10}	4.9666	T_{10}	4.3136	S_{10}	4.8451	T_{10}	4.2123

Table S2. Absorption and emission peak positions of compounds in different solvents.

Solvents	$f(\varepsilon,n)$	N-TBPMCN		
		λ_a (nm)	λ_f (nm)	ν_a-ν_f (cm^{-1})
**Hexane**	0.0012	347	403	4004.54802
Triethylamine	0.048	347	408	4308.63988
Butylether	0.096	347	412	4546.59914
Isopropyl ether	0.145	347	417	4837.62846
Ether	0.167	347	417	4837.62846
Ethyl acetate	0.200	347	426	5344.2654
Tetrahydrofuran	0.210	347	429	5508.42049
Dichloromethane	0.217	347	433	5723.75558
Dimethylformamide	0.276	347	436	5882.66399
Acetone	0.284	343	435	6166.0132
Acetonitrile	0.305	342	436	6303.98627

*The abbreviations: f: polarity factor of solvents; ε: dielectric constant; λ_a: absorption wavelength; λ_f: emission wavelength; v_a: wave number of the absorption spectrum; v_f: wave number of the emission spectrum.

Table S3. PLQY of N-TBPMCN in different solvents.

Solvents	Hexane	Ether	THF	Acetonitrile
PLQY (%)	55	73	14	1

S-III Illustrations

3.1. General information

A Bruker AVANCE 500 spectrometer was used for the ^{1}H NMR and ^{13}C NMR measurement at 298 K. The deuterated dimethyl sulfoxide (d-DMSO) and deuterated chloroform (d-$CHCl_3$) as solvents and tetramethylsilane (TMS) as a standard were purchased from J&K Scientific. The Flash EA 1112, CHNS-O elemental analysis instrument was used to characterize these compounds. The differential scanning calorimetry (DSC, DSC Q100) PerkinElmer thermal analysis system was set at a heating rate of 10 °C min^{-1} and a nitrogen flow rate of 80 mL min^{-1}. The thermogravimetric analysis (TGA, TA 2050) was set at a scanning rate of 10 °C min^{-1} in a nitrogen flow. The matrix-assisted laser desorption ionization time-of-flight mass spectrometry（MALDI-TOF MS）was operated on the AXIMA-CFRTM plus instrument. A BAS 100W Bioanalytical Systems was used to record cyclic voltammetry (CV). The glass carbon disk (Φ = 3 mm), platinum wire and Ag/Ag$^+$ electrode were used as working electrode, auxiliary electrode and reference electrode, respectively. The calculation mainly relied on PowerLeader cluster instrument.

3.2. Lippert–Mataga model

The Lippert–Mataga model can describe the relationship between excited state dipole moment and the solution polarity. Based on the related dipole moment of the solvent, the dipole moment of the excited state can be calculated according to the following equation:

$$hc(\nu_{\mathrm{a}} - \nu_{\mathrm{f}}) = hc(\nu_{\mathrm{a}}^0 - \nu_{\mathrm{f}}^0) - \frac{2(\mu_e - \mu_g)^2}{\mathrm{a}^3} f(\varepsilon, n) \quad (1)$$

the f is the orientational polarizability of solvents, μ_e is the dipole moment of excited state, μ_g is the dipole moment of ground state; a is the solvent cavity (Onsager) radius, ε and n are the solvent dielectric and the solvent refractive index, respectively.

3.3. Relative PLQY measurement

The PLQY in solution is with a 0.1 mol/L quinine sulfate aqueous solution for reference. The PLQY of quinine sulfate aqueous solution is 0.546, and the PLQY of the sample can be calculated by the following equation：

$$\mathrm{QY}_s = 0.546 \cdot \frac{I_s}{I_q} \cdot \frac{A_q}{A_s} \quad (2)$$

the QY_s is the relative quantum yield, the I_s and I_q are the area of sample and quinine sulfate aqueous solution emission spectrum, and the A_s and A_q are the absorbance of sample and quinine sulfate aqueous solution at 365 nm.
